# New Insights into Bile Acids Related Signaling Pathways in the Onset of Colorectal Cancer

**DOI:** 10.3390/nu14142964

**Published:** 2022-07-20

**Authors:** Cristiana Caliceti, Angela Punzo, Alessia Silla, Patrizia Simoni, Giulia Roda, Silvana Hrelia

**Affiliations:** 1 Department of Biomedical and Neuromotor Sciences, Alma Mater Studiorum, University of Bologna, 40126 Bologna, Italy; 2 Interdepartmental Centre for Renewable Sources, Environment, Sea and Energy (CIRI FRAME), Alma Mater Studiorum, University of Bologna, 40126 Bologna, Italy; 3 Biostructures and Biosystems National Institute (INBB), 00136 Rome, Italy; giuliaroda@gmail.com; 4 Department of Chemistry “Giacomo Ciamician” Alma Mater Studiorum, University of Bologna, 40126 Bologna, Italy; angela.punzo2@unibo.it; 5 Department for Life Quality Studies, Alma Mater Studiorum, University of Bologna, 40126 Bologna, Italy; alessia.silla2@unibo.it (A.S.); silvana.hrelia@unibo.it (S.H.); 6 Department of Medical and Surgical Sciences, Alma Mater Studiorum, University of Bologna, 40138 Bologna, Italy; patrizia.simoni@unibo.it

**Keywords:** colorectal cancer, bile acids, FXR, VDR, PXR/SXR, TGR5

## Abstract

Colorectal cancer (CRC) ranks as the second among the causes of tumor death worldwide, with an estimation of 1.9 million new cases in 2020 and more than 900,000 deaths. This rate might increase by 60% over the next 10 years. These data are unacceptable considering that CRC could be successfully treated if diagnosed in the early stages. A high-fat diet promotes the hepatic synthesis of bile acids (BAs) increasing their delivery to the colonic lumen and numerous scientific reports correlate BAs, especially secondary BAs, with CRC incidence. We reviewed the physicochemical and biological characteristics of BAs, focusing on the major pathways involved in CRC risk and progression. We specifically pointed out the role of BAs as signaling molecules and the tangled relationships among their nuclear and membrane receptors with the big bang of molecular and cellular events that trigger CRC occurrence.

## 1. Introduction

Colorectal cancer (CRC) ranks as the fourth most common cancer and the second cause of cancer in men and the third in women. The International Agency for Research on Cancer (IARC) estimated 1.9 million new cases in 2020, with more than 900,000 deaths, and this rate might increase by 60% over the next 10 years [1]. These data are unacceptable considering that CRC could be successfully treated if diagnosed in the early stages. To date, the guaiac-based test of fecal occult blood (FOBT), although not very sensitive, is used in most CRC screening programs, eventually followed by endoscopic assessment and prophylactic surgical resection in cases of high-grade dysplasia [2].

This rapid growth may reflect both the increased lifespan, as well as genetic predisposition, such as positive family history (almost 20% of all patients) and other genetic syndromes, such as Lynch and Gardner syndromes, and familial adenomatous polyposis (FAP) [3], as well as environmental factors.

The cancer gradual progression offers an opportunity to detect it before the malignant changes; studies have shown that over 90% of all CRC results from colon–rectal adenomas and just the metastatic phases are related with a high mortality rate and a 5-year survival of below 10% [4].

The CRC pathogenesis is a multi-step process that involves a series of events from adenoma to carcinoma with the histological transition of the normal mucosal epithelium. The stepwise accumulation of genetic and epigenetic alteration leads to a generalized disorder of stem and epithelial cells replication and differentiation, thus causing aberrant proliferation of cells within crypts that may progress to becoming adenomas with different grades of dysplasia and may even become more invasive leading to carcinoma [5].

There are several mechanisms underlying tumorigenic development, such as the aberrant activation of oncogenes and the inactivation of tumor suppressors. In the hereditary CRC forms, there has been frequently reports of a mutation in the adenomatous polyposis coli (APC) gene, which plays a key role in the Wnt/B-catenin signaling pathway, and a mutation in the family of mismatch repair genes (MMR), which preserves genomic integrity. Instead, in sporadic CRC, which accounts for nearly 85% of all CRC cases, it has been identified a plethora of mutations, which can lead the oncogenic events. Signaling pathways, such as Wnt/β catenin, the mitogen-activated protein kinase (MAPK), the phosphatidylinositol 3-kinase (PI3K)/protein kinase B (AKT), the transforming growth factor-β (TGF-β), and Notch, have been frequently reported to be dysregulated in the CRC [6,7]. These pathways are closely interconnected, setting up an intricate network that supports the tumorigenesis and makes CRC treatment very complex.

There is a strict correlation between an unhealthy diet, dysbiosis, bile acids (Bas) synthesis and metabolism, and CRC; indeed, epidemiological studies showed that over 90% of gastric and colonic cancers could be imputable to diet [8]. Bas hepatic synthesis from cholesterol displays a marked diurnal rhythm, which is driven by feeding and fasting, as well as nutrient status, maintaining the metabolic homeostasis [9,10].

BAs are cholesterol end-product derivatives with detergent-like properties; a high-fat consumption increases their synthesis into the liver and their delivery to the large bowel where they are metabolized by the microbiota into secondary Bas, which are products with potential inflammatory and tumorigenic activity. Moreover, different transport rates in jejunum and ileum have been reported [11].

When BAs are at high concentrations, they self-aggregate to form micelles that could cause cell membrane injury resulting in the loss of the intestinal epithelium integrity. This damage consequently induces repair mechanisms involving inflammatory processes and hyperproliferation of undifferentiated cells [12]. These reactions could trigger a cell transition (epithelial to mesenchymal transition, EMT) into a precancerous state and are considered an early priming step in colorectal tumorigenesis. Moreover, BAs at high concentrations modify microbiota composition, leading to dysbiosis, thanks to their detergent properties [13].

During the past years, the role of BAs, not only as detergents for dietary lipids and vitamin absorption but also as signaling molecules that regulate the activity of lipid and glucose metabolic pathways, has been well established [14]. Several studies in the last decades have unveiled new functions of BAs as signaling molecules involved in CRC development. The bile acid-activated receptors farnesoid X receptor (FXR), pregnane X receptor (PXR), vitamin D receptor (VDR), and G protein-coupled bile acid receptor (TGR5) play critical functions not only in the regulation of lipid absorption and energy homeostasis but also in the onset of different pathologies, including CRC.

Herein, the role of BAs as signaling molecules involved in principal mechanisms underlined the CRC initiation and progression is discussed. We reviewed in deep the physicochemical and biological characteristics of BAs, focusing on the concept of detergency and lipophilicity that are often confused and misinterpreted. We described the major signaling pathways dysregulated in CRC risk and progression, specifically pointing out the role of BAs as signaling molecules and the tangled relationships among their nuclear and membrane receptors with the big bang of molecular and cellular events that trigger CRC occurrence.

## 2. Search Strategy (Methods)

The purpose of this literature review is to satisfy the following research questions: (i) What are the physicochemical properties of BAs? (ii) What are their main functions in our body? (iii) Is there an association between dysregulated BAs production/excretion and CRC? (iv) Which are the most relevant signaling pathways involved in CRC onset? (v) Is there any overlapping changes in the BA composition in people suffering from CRC? (vi) Which are the BA receptors, and what is their role in modulating the most relevant signaling pathways involved in CRC onset?

**Figure 1:**
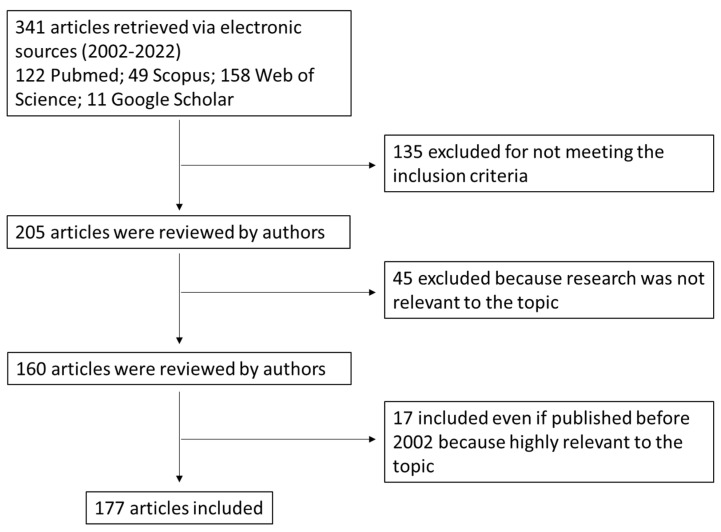
Graphical scheme of bibliographic search.

The literature search was run on the electronic databases Scopus, PubMed, Google Scholar, and Web of Science for peer-reviewed research articles until June 2022 published in English (Figure 1). The search started initially by combining the following search and MeSH terms: bile acids, colon cancer, signaling pathways, and bile acid receptors. Numerous articles were obtained, some of which were not directly related to the field of interest; therefore, the screening, study selection, and data extraction were undertaken by four independent authors. Disagreement was resolved by debate and, if required, by a fifth independent author. The papers included in our search had to fulfil the listed inclusion criteria below:

- Discussing BAs synthesis and metabolism, physicochemical properties and BA receptors in the gut;
- Including findings from human and animal studies (if relevant) with the support of preclinical data related to the role of BAs in CRC onset;
- Published in peer-reviewed journals;
- Available in full-text;
- Written in English.
- Published within the time frame of 2002–2022. Papers published before 2002 were included if they were relevant in the field.

## 3. Bile Acids: An Overview

BAs are the end products of the catabolism of cholesterol, exclusively produced in hepatocytes. They are the major constituents of bile, a duodenal fluid, acting both as physiological detergents with phospholipids (at an mM micellar concentration) essential to dietary lipid absorption [15] and as signaling molecules in cells (at very low concentration) implicated in distinct pathways and implicated in the control of their homeostasis and in some metabolic regulations, such as glucose and energy homeostasis, mainly in hepatic and intestinal cells [16].

**Figure 2:**
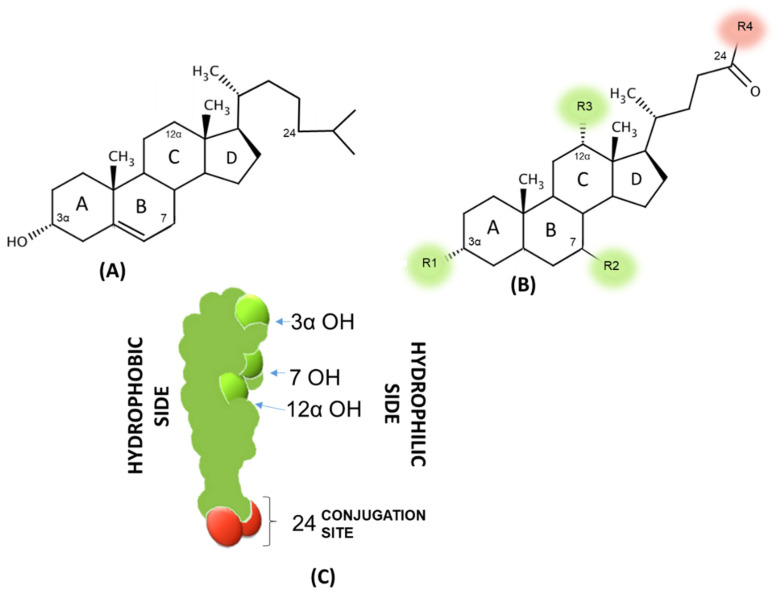
(**A**,**B**) General structures of cholesterol and bile acids. (**C**) Space-filling model of a BA. All three hydroxyl groups and the carboxyl group are faced to one side of the carbon skeleton to form a hydrophilic face, which is opposite to the hydrophobic face of the carbon skeleton.

Concerning their chemical structure, common BAs have 24 carbon atoms and, for this reason, are abbreviated as C24 BAs. The steroid nucleus is composed of four fused carbon rings consisting of three 6-carbon rings and one 5-carbon ring [17] (Figure 2). The transition of cholesterol into BAs involves a total of 17 distinct enzymes that are located in different cellular compartments of pericentral hepatocytes, such as cytosol, endoplasmic reticulum, mitochondria, and peroxisomes [18]. These enzymes catalyze the changes of the steroid ring and the oxidative cleavage of three carbons from the cholesterol side chain, thus forming C24 BAs. 

### 3.1. Bile Acid Synthesis and Metabolism

**Figure 3:**
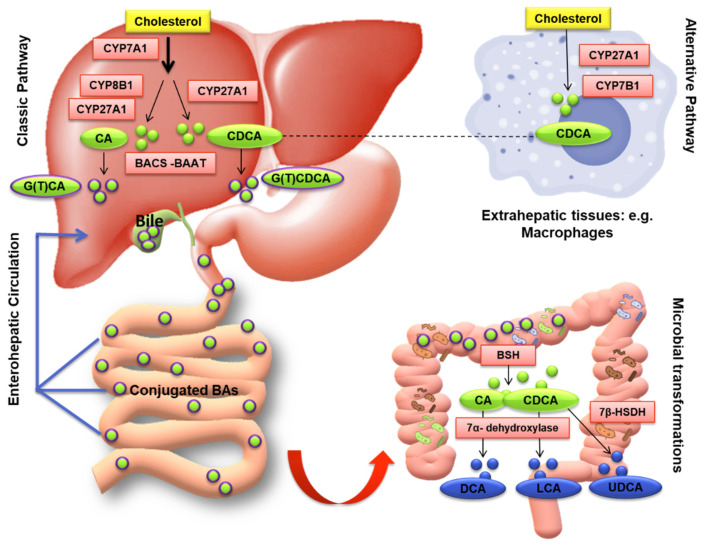
Schematic representation of pathways involved in BAs synthesis and metabolism. BAs synthesis takes place from cholesterol in the liver or extrahepatic tissues (i.e., macrophages), through the classical and the alternative pathway, respectively. Cytochromes CYP7A1, CYP27A1, and CYP8B1 are involved in the classical pathway, while cytochromes CYP27A1 and CYP7B1 are in the alternative pathway. Both pathways lead to the production of the primary BA, chenodeoxycholic (CDCA), while cholic acid (CA) is just synthesized in the hepatic classical pathway. Of the 2 major biosynthetic pathways, the classical (CYP7A1) pathway is more important in adult humans in a quantitative point of view. Primary BAs neo-synthesized are conjugated with glycine or taurine CA and CDCA are conjugated with glycine or taurine by bile acid–CoA synthase (BACS) and bile acid–CoA- amino acid N-acetyltransferase (BAAT) to produce the GCA, TCA, GCDCA, TCDCA and stored in the gallbladder. In presence of CCK, they are released into bile in the duodenum where they participate in the digestion of fats. In the ileum, under the action of intestinal bacteria, BAs are deconjugated and converted into secondary BAs deoxycholic acid (DCA) and lithocholic (LCA). In the colon, most unconjugated BAs are reabsorbed and transported to the liver by the portal vein, to participate in a new round of digestion (enterohepatic circulation). Excess of BAs is eliminated by the feces. The figure is original and created with BioRender.com.

There are two major pathways for BAs biosynthesis: a classic or “neutral” pathway and an alternative or “acidic” pathway (Figure 2). In the classic or neutral pathway, so named because its intermediate metabolites are neutral sterols, steroid ring modification precedes side-chain cleavage; on the other hand, in the alternative or acidic pathway these two steps are reversed. The classic pathway begins with the hydroxylation of cholesterol at C-7 (on the steroid nucleus), which is catalyzed by cholesterol 7α-hydroxylase (CYP7A1), the only rate-limiting enzyme of the pathway, and synthesizes two primary BAs: cholic acid (CA; 3α,7α,12α-trihydroxy-5β-cholan-24-oic acid) and chenodeoxycholic acid (CDCA; 3α,7α-dihydroxy-5β-cholan-24-oic acid) in the human liver [19]. A microsomal sterol 12α-hydroxylase (CYP8B1) is required for CA synthesis; without this enzyme, the resulting product is just CDCA. Finally, sterol 27-hydroxylase (CYP27A1) catalyzes the steroid side-chain oxidation reaction (the terminal methylene group of the side chain) (Figure 3). Interestingly, 7α-hydroxycholesterol (7α-HC) is one of the most representative cholesterol oxidation products that contribute to the progression of chronic/degenerative diseases, such as neurodegenerative, atherosclerosis, diabetes, osteoporosis, etc., due to its pro-inflammatory, pro-apoptotic, cytotoxic, carcinogenic, and mutagenic effects [20].

The alternative (acidic) pathway starts with hydroxylation at C-27, catalyzed by CYP27A1, which synthesizes oxidized sterols, followed by oxysterol hydroxylase (CYP7B1), a microsomal enzyme specific for the synthesis of CDCA. In humans, the acidic pathway contributes only a small fraction (3–18%) of total newly synthesized BAs but may be quantitatively important in BAs biosynthesis in patients with liver diseases and neonates. Notably, the liver is the only organ where the classic BAs synthesis pathway takes place while the alternative pathway can occur in various body parts besides the liver, such as in macrophages, brain, adrenal glands, and other peripheral tissues [18] (Figure 3).

The final step of BAs synthesis occurs in peroxisomes and involves the conjugation (amidation) of the terminal side-chain carboxylic acid with the amino acids glycine and/or taurine, usually in a ratio of 3:1, respectively, in humans, carried out by the bile acid-coenzyme A (CoA) synthase (BACS, also known as SLC27A5) and bile acid-CoA: amino acid N-acyltransferase (BAAT) (quite different in animals, i.e., in rodents the taurine amidation is the main step) [21]. The conjugation is a fundamental prerequisite for BAs secretion into bile, since most of the unconjugated BAs are too lipophilic to be excreted and conjugation largely decreases the lipophilicity (Table 1) [20]. The conjugated BAs have a pKa value lower than unconjugated BAs, 3.9 for glycine and <1 for taurine conjugated in respect to 5 for unconjugated BAs; this implies that they are ionized at a low pH, for instance, taurine-conjugated BAs are always ionized in all biological fluids. The newly synthesized conjugated BAs are released from the liver into the bile canaliculus via the canalicular bile salt export pump (BSEP), and then stored in the gallbladder as bile salts in the bile, a micellar solution containing phospholipids and cholesterol that is 10–50 time more concentrated of BAs than intestinal milieu, awaiting hormonal signals to empty after a meal (Figure 3). BAs conjugation renders them less hydrophobic, helps to keep cholesterol in the solution at physiological pH, prevents Ca^2+^ precipitation and passive absorption, and makes them resistant to pancreatic carboxypeptidases cleavage [17], and thus ready to exploit their physiological functions in the gut. Indeed, after food intake, BAs are excreted into the gastrointestinal tract acting as detergents to promote the absorption of lipophilic nutrients, such as dietary fats, steroids, and liposoluble vitamins, drugs, and other compounds. Thus, to simplify, taurine or glycine conjugation makes BAs non-absorbable and indigestible in the proximal small intestine where most lipid absorption takes place.

**Figure 4:**
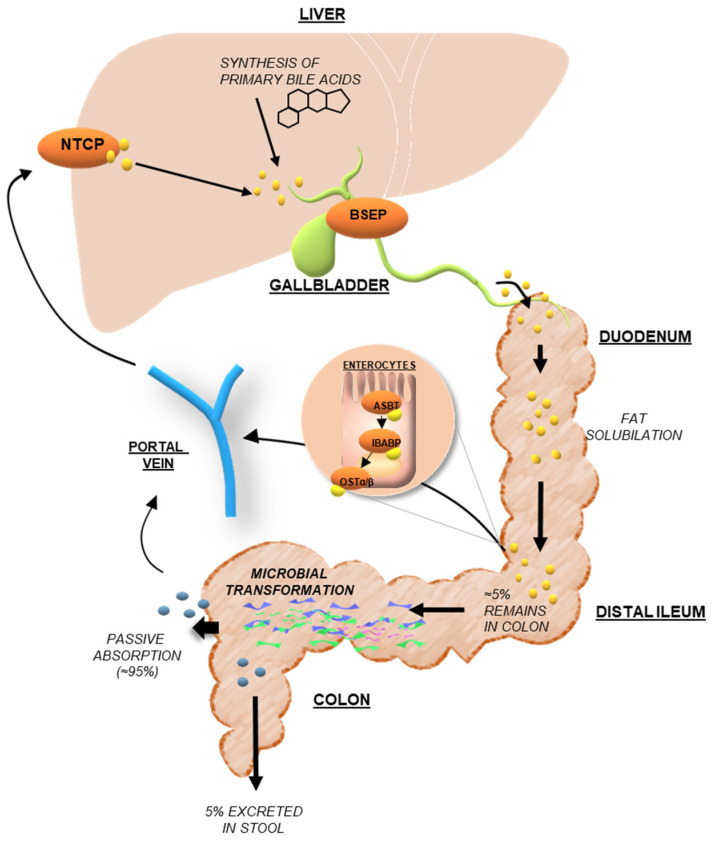
Representation of BAs enterohepatic circulation. After a meal, conjugated BAs are released from the gallbladder to the duodenum. When reaching the distal ileum, conjugated BAs are retrieved by ASBT at the luminal membrane, addressed to the basolateral membrane of the ileal enterocyte by the cytosolic transporter IBABP, and then released in the portal blood via the OSTα/β transporter to come back to the liver and be taken up by the NTCP transporter and complete the enterohepatic circulation. Via the canalicular bile salt export pump (BSEP), BAs are then stored in the gallbladder as bile salts in the bile. Secondary unconjugated BAs are passively reabsorbed in the colon. The figure is original and created with BioRender.com.

After each meal, the gallbladder evacuates its contents in response to the cholecystokinin (CCK) hormone, promoting the release of BAs in the small intestine thus enables the emulsification and absorption of lipids and liposoluble vitamins [26]. After a meal, more than 80% of the stored conjugated BAs are expelled into the proximal small intestine [27]. Following lipid absorption, conjugated BAs are reabsorbed in the distal ileum by the apical sodium-dependent bile acid transporter (ASBT), a symporter carrier able to co-transport two sodium ions with one molecule of BA. Indeed, since they are in ionized (deprotonated) form in the alkaline intestinal milieu (A-) but hydrophilic (with a low LogP), they cannot be passively absorbed across the enterocyte membrane (Table 1) [20]. On the contrary, unconjugated BAs, even if in ionized form al physiological pH, can be passively absorbed as highlighted by the LogP of the ionized form, which is greater than one (Table 1). BAs are then bound to the ileal bile acid-binding protein (IBABP), which is involved in the BAs transport across the enterocyte to the basolateral membrane, where in turn the enteric organic solute transporter α and β (OSTα–OSTβ) complex mediates the transport into the portal vein. Once they enter the hepatic portal circulation, they are addressed to the liver; so, removed from the blood (70–90%). Upon entering the bloodstream, conjugated bile salts bind to plasma proteins (i.e., human serum albumin, HSA) and turn back to the liver where they are efficiently cleared from the circulation by active transporters (NTCP) on the sinusoidal membrane of hepatocytes and rapidly secreted into bile [28] (Figure 4). Therefore, low systemic concentrations (2–4 μM) of BAs are usually observed in respect with high hepatic portal concentrations (60–80 μM), unless a liver disease is present. Primary deconjugated BAs can be reabsorbed, return to the liver, and re-conjugated during transit to hepatocytes [29].

Once reaching the liver again, conjugated BAs are actively transferred from the hepatocytes into the bile, inducing bile flow and cholesterol and phospholipids release. This recovery process is also known as enterohepatic circulation (EHC) [30]. Therefore, the newly synthesized BAs can be released again, 20–40 times during digestion. This process of storage and secretion requires a system of tightly regulated transporters allowing not only the BAs metabolism homeostasis but also the control of cholesterol maintenance from which they originated (Figure 4).

Although ileal BAs absorption is a very efficient process (~95%), approximately 400–800 mg of BA salts escape the daily EHC [31]. The BAs loss is restored by a new synthesis from cholesterol by a feedback mechanism that ensures their constant level in EHC. The unabsorbed fraction undergoes several bacterial biotransformations in the large bowel to produce secondary hydrophobic BAs: deoxycholic acid (DCA; 3α,12α-dihydroxy-5β-cholanoic acid), lithocholic acid (LCA; 3a-hydroxy-5β-cholanoic acid), and the 7β epimer of CDCA ursodeoxycholic acid (UDCA; 3α,7β-dihydroxy-5β-cholanoic acid) could be formed, sometimes called the tertiary BA (Figure 2 and Table 1). During their intestinal transit, BAs face populations of facultative and anaerobic bacteria of relatively low numbers and diversity in the microbiota placed in the small intestine (Figure 3 and Figure 4). Moreover, a small quantity of BAs may spill over into the systemic circulation, reabsorbed when passing through the renal tubules in the kidney, and then redirected to the liver through systemic circulation (Figure 4) [32].

The major BA modifications in the human colon include the first deconjugation, then 7α-dehydroxylation limited to the unconjugated BAs, and the oxidation/epimerization of hydroxyl groups at C-3, C-7, and C-12. Primarily, the conjugated ones are hydrolyzed by bile salt hydrolases (BSH), a cytoplasmic enzyme from the microbiota, such as Bacteroides, Clostridium, Lactobacillus, Bifidobacterium, and Listeria, which can catalyze the hydrolysis of a conjugated BAs into its unconjugated form and free amino acid moieties [33]. Conjugate BAs are not directly metabolized to secondary BAs and should be firstly deconjugated by BSH, which largely functions as a detoxification mechanism to reduce the bile salts levels in the colonic environment (Figure 3 and Figure 4). Then, anaerobes of the genera Bacteroides, Clostridium, Eubacterium, Lactobacillus and Escherichia convert the unconjugated primary BAs into the secondary BAs LCA and DCA through 7α dehydroxylation by CYP7A1 [34,35]. Lately, six new microbial enzymes involved the conversion of CA into DCA have been identified, establishing a complete roadmap from a primary BA to its secondary derivatives [36].

Thus, the secondary BAs, DCA and LCA, formed from CA and CDCA, respectively, by anaerobic gut bacteria, can then be passively absorbed from the large bowel, and mostly secreted in the feces (Figure 4).

In humans and other species with gallbladders, BAs have been discovered to cross the entire EHC (i.e., liver → bile → intestine → portal blood → liver) approximately 12 times/day, while animals without gallbladders (e.g., horses, rodents or dogs having undergone cholecystectomy) maintain a much smaller BAs pool, yet they cycle it more often (approximately 33 times/day). Around 1% of the BAs pool passes into the large intestine and is lost with each cycle via fecal excretion; only about 12% of the pool requires a replacement each day by new bile acid synthesis in the liver, approximately 500–600 mg/day [13].

### 3.2. Physicochemical Properties of Bile Acids

The most important physicochemical properties involved in BAs metabolism and toxicity are lipophilicity, i.e., the tendency to distribute in a lipid domain from an aqueous solution described by the octanol/water partition coefficient (LogP) and the detergency, i.e., their self-aggregation to form micelles also with phospholipids and, therefore, acting as a natural surfactant toward ordered lipid structures and cells described by the critical micellar concentration (CMC). To note, BAs that tend to stay more in the non-polar phase (1-octanol) are defined as hydrophobic (with a logP value > 0). The two properties are not correlated since some lipophilic BAs are not detergent and vice versa since the detergency is related to a proper hydrophilic/hydrophobic balance, which drives the formation of the micelles via BA back-to-back interactions of the hydrophobic side (Figure 2C); any reduction in this area due to the presence of hydroxyls oriented toward this side (7 or 12β and 6 α) increases the CMC and, therefore, reduces the detergency. Among the naturally occurring BAs in humans, the more lipophilic BA is LCA followed by DCA, CDCA and UDCA with similar hydrophobicity (2 hydroxyl groups), and CA, which is the less hydrophobic (3 hydroxyl groups). The conjugation with glycine and taurine highly reduces the hydrophobicity of the unconjugated BAs by the effect of the amide bond at the C24 position. The drastic reduction in LogP from 1–3 to almost negative values accounts for the poor intestinal absorption of the conjugated BAs while simultaneously an efficient one by the unconjugated BAs (Table 1).

The detergency behaves quite differently, and the more detergent BAs are the DCA and CDCA followed by CA. UDCA, despite a similar hydrophobicity with DCA and CDCA because of a hydroxyl in 7 β positions oriented toward the hydrophobic side, is much less detergent and, therefore, safer toward membrane damage as the opposite occurs for CDCA and DCA. Surprisingly, UDCA acts as protector against membrane damage induced by CDCA and DCA. Conjugation slightly increases the detergency as the CMC is reduced in respect to the corresponding unconjugated BAs, and this is apparently in contrast with the fact that they are less lipophilic, but the side chain modification mainly acts to the hydrophobicity due to the amide bond but does not affect the self-association process. To summarize, BAs could be hydrophobic but poor detergents, and this implies that the molecule could cross membranes without major damage but just accumulate in the lipid domain. Other BAs did not come across as membranes being hydrophilic, but at a given concentration (above CMC) became cytotoxic, since they promote the solubilization of the main lipid constituents of plasma membranes.

LCA and its conjugates behave quite differently from the other BAs; the water solubility of LCA is less than 0.1 µM and does not reach a concentration to form micelles even when ionized. LCA with a very high LogP could accumulate in cells and precipitate. GLCA has a higher solubility than unconjugated LCA (around 40 uM) but is still lower than that needed to self-aggregate. For these compounds, only hydrophobicity plays a physio-pathological role [19,20].

Another important physicochemical property is the acidity constant pKa. The unconjugated BAs independently from the steroid hydroxyl groups have a pKa value of five, and this means that at a physiological pH they are fully ionized. In addition, the glycine-conjugated BAs present a lower pKa of 3.9 and are, therefore, ionized even at a lower pH with respect to unconjugated BAs while the taurine-conjugated BAs are always ionized independently from the pH of the medium [37]. All these data showed that unconjugated BAs even ionized are still lipophilic and absorbed by passive diffusion in the intestine, while conjugated BAs are not passively absorbed.

The 3-, 7- or 12-hydroxyl groups of BAs can undergo oxidation through the activity of hydroxysteroid dehydrogenases (HSDHs) of intestinal bacteria, such as Bacteroides, Eubacterium, Clostridium, Escherichia, Eggerthella, Peptostreptococcus and Ruminococcus, leading to numerous oxo-BAs structures with a different arrangement in position and orientation of hydroxyl groups. Epimerization of BAs hydroxyl groups is a reversible conversion in stereochemistry from the α to the β configuration (or vice versa), with the generation of a stable oxo bile acid intermediate [38]. For instance, the CDCA epimerization represents the origin of UDCA, the 7-β epimer, which is a minor constituent of the human BAs pool.

Consequently, the microbial metabolism of the BAs results in a raised variety and a more hydrophobic BAs pool, and for this reason, a variation of the intestinal microbiota composition is reflected in the BAs biological modification [39]. The role of oxo BAs remains undefined and unclear because this reaction is not favored in terms of energy. Indeed, the formation of more hydrophobic compounds is quite uncommon not only for energy expenditure but also for excretion purposes. Roda et al. suggested that since the oxo BAs analyzed in feces samples through HPLC-ESI-MS/MS were metabolic derivatives of secondary BAs (DCA, LCA and UDCA), which are usually the only BAs present in the colon, this extensive metabolism to produce oxo analogues might be a way to limit the secondary BAs level in the colon tract, since the damage due to the toxicity and carcinogenicity of these derivatives should be lower than that of their precursors [38]. In the same context, Ridlon et al. suggested that the oxo BAs could represent signaling molecules that influence other microbes or could also alter host physiology in a way that benefits the bacterial organism itself [40].

DCA levels can differ from 1% to over 50% as the human liver is unable to 7a-hydroxylate DCA to the primary bile acid CA, thus it accumulates in the bile acid pool (LCA to a much lesser extent) as a result of passive absorption through the colonic mucosa [41]. As the amount of DCA is increased in the BAs pool, there is an increase in the detergent activity and cytotoxicity in mammalian cells, which can lead to inflammation, enhanced epithelial apoptosis, and cytokine accumulation. LCA is the only natural monohydroxy bile acid and is one of the major components of fecal BAs (DCA/LCA ratio 1:2). LCA can bind the vitamin D receptor (VDR) in colonic cells, carrying out vitamin D functions, such as the regulation of calcium metabolism calcium and adaptive immunity [42,43], but also induces expression of CYP3A1, a cytochrome P450 enzyme involved in its detoxification. On average, 50% of the DCA is absorbed and enters the EHC, where it is conjugated in the liver and secreted in the bile. On the contrary, LCA is fairly insoluble and just a small amount is reabsorbed [44]. Absorption occurs by passive flip-flop of the nonionized BAs molecule across the lipid bilayer. Indeed, the deconjugation and 7α-dehydroxylation of BAs increase their pKa and hydrophobicity, allowing recovery by passive absorption across the colonic epithelium. The resulting BAs, which are not absorbed in the colon or previously in the small intestine, are lost in the feces and represent 5% of the total BAs pool. Thus, the BAs pool, which goes into the EHC, is composed of about 30 to 40% each of CA and CDCA, about 20 to 30% of DCA, and less than 5% of LCA [41]. The very low amount of LCA that recirculates to the liver can be sulfo-conjugated at the 3-hydroxy position by sulfotransferase (SULT2A1) and excreted back into bile. The resultant 3-sulfo-LCA is lost in feces and does not usually remain in the systemic circulation; however, 3-sulfo-LCA glycine and taurine conjugates can be deconjugated and to some extent desulfated by intestinal bacteria [45]. Indeed, the sulfation of BAs due to the presence of a negative charge decreases their intestinal absorption, increases their solubility, and enhances their urinary and fecal excretion. Furthermore, BA sulfates are also less toxic than their unsulfated counterparts. Thus, sulfation is the major pathway for the detoxification of extremely hydrophobic BAs in humans. LCA sulfation was the first to be discovered, however, all endogenous BAs may be found in the sulfated form in various proportions [46].

## 4. Dysregulated Signaling Pathways in Colorectal Cancer: The Emerging Role of Bile Acids

In the intestine, the largest barrier against the external environment is represented by the epithelial surface. In most adult mammals, epithelial cells are renewed every 2 to 5 days to counteract intestinal damage; it is the fastest self-renewing tissue in mammals [47]. Renewal and patterning coordination of the intestinal epithelium is controlled by intestinal stem cells (ISCs), which reside at the bottom of intestinal crypts and respond to tight regulation to fine-tune the balance between proliferation and cell fate specification [48].

CRC is a multifactorial disease involving genetic, environmental, and lifestyle risk factors [49]. Although the hereditary components strongly affected CRC, most cases are sporadic and gradually develop over several years in a step-wise manner known as the adenoma–carcinoma sequence [50]. The mechanism behind CRC development is a complex multistage process, primarily involving sequential mutational events occurring along with the progression of cancer [50]. One of the major etiological factors of CRC is represented by an increased intestinal inflammatory state also carried by defensive cells, such as T-lymphocytes, macrophages, natural killer cells, and other cells, which may have a key function in tumor development, [51] also leading to dysbiosis. These circumstances, in turn, can result in a greater alteration of intracellular signaling pathways, initiation, and increase in mutations in tumor cells that promote active and rapid proliferation and protect the cancer cells from apoptosis. Consequently, CRC cells lose the epithelial phenotype and concomitantly gain the mesenchymal phenotype, thus undergoing an epithelial–mesenchymal transition (EMT) allowing them to metastasize [52].

The dysregulation of several signaling pathways involved in fundamental cellular processes, including cell proliferation, differentiation, angiogenesis, apoptosis, and survival, (e.g., Wnt, TGF-β, MAPK, PI3K/Akt, EGFR, and Notch signaling pathways) are reported as key factors in CRC onset. Various genes, associated with the signaling pathways previously mentioned, have been frequently reported to be dysregulated due to mutations or the altered function of their products in the CRC, such as APC, RAS, and RAF [50,51]. Moreover, since the role of crosstalks between signaling pathways in CRC is remarkable, a brief description and connections between these pathways may be fundamental to better understand the possible mechanisms behind bile acids receptors regulation.

### 4.1. The Molecular Characteristics of Colorectal Cancer: Implications of the Major Signaling Pathways

Vogelstein and coworkers firstly proposed a multistep genetic model that has come to serve as a paradigm for CRC [50]. The Wnt/β-catenin cascade plays the single most dominant role in controlling the epithelial cells’ fate in the intestine. Indeed, an early transformation of epithelial cells involves a mutation in the adenomatous polyposis coli (APC) or Axin2 (in 70% of colorectal adenomas), followed by a long-term activation of β-catenin [53]. Indeed, in humans the most common driver mutation for colorectal carcinoma is related to a mutation in the tumor suppressor gene APC, leading to the inactivation of APC and activation of the Wnt signaling pathway, with a consequent stabilization of β-catenin and its translocation to the nucleus.

Moreover, the activation of the K-Ras/RAF/MAPK pathway due to additional mutations results in the growth of a small adenoma to a clinically significant size (>1 cm) [54]. The further acquisition of inactivating mutations of the transforming growth factor-β (TGF-β) confers additional malignant features to adenoma cells. Other mutations in the onco-suppressor p53 finally result in the subsequent malignant transformation to adenocarcinoma [50,53]. All these mutations occur as a ‘big bang’ event in a limited subset of epithelial (stem) cells and seem to drive malignant phenotypes by rewiring cell-autonomous signaling in epithelial cells and promoting epithelial proliferation and self-renewal [55]. Indeed, for instance, the loss of APC hyperactivates β-catenin signaling, whereas K-Ras point mutations hyperactivate the RAF–MAPK cascade. Recently, a mathematical model of CRC initiation has been developed in which the loss of tumor suppressors APC and p53 and the gain of the K-Ras oncogene were defined as the key players of the complex network of premalignant mutational genotypes on the way to CRC [56]. Van Neerven et al. and Flanagan et al. pointed out that several genes associated with Wnt pathway inhibition, mainly the gene Notum, exhibited higher expression in cells with an APC mutation than in wild-type cells [57,58] its expression increases with ageing, as well as the incidence of CRC [59,60].

The epidermal growth factor receptor (EGFR) pathway has also been associated with the CRC progression since the binding of its ligand EGF stimulates downstream signaling cascades, such as MAPK, PI3K/Akt, p53, and signal transducer and activator of transcription (Stat)-3 signaling pathways [61], which are linked to tumor cell proliferation, survival, angiogenesis, invasion, and metastasis [62]. However, it is well known that the benefit of the anti-EGFR therapy is restricted to around 40% of the patients who are K-Ras and RAF wild type [63]. In CRC, the PI3K/Akt dysregulation appears to primarily occur due to mutations in the PIK3CA gene [64] or in the phosphatase and tensin homolog (PTEN) gene, which is a negative regulator of PI3K. Specifically, the PIK3CA mutation and PTEN dysregulation were found in about 15–18% and 46%, respectively, and are significantly associated with K-Ras mutations [65,66].

The oncogenic role of PI3K/Akt pathways is also linked to crosstalk with two other pathways. Firstly, PI3K up-regulates β-catenin signaling. Indeed, it may inactivate Glycogen synthase kinase 3 β (GSK3B), thus blocking the formation of β-catenin-destroying-complex and promoting the β-catenin accumulation, triggering the Wnt downstream signaling and resulting in the promotion of neoplastic events [67]. Secondly, Akt may regulate the transcriptional activity of the nuclear factor-κB (NF-κB) by inducing phosphorylation and subsequent degradation of the Inhibitor of κB (IκB). Indeed, several cancers show constitutively elevated levels of NF-κB activity, and it has been found that the inhibition of NF-κB through the over-expression of IκB strongly interferes with oncogenic processes induced by PI3K/Akt [68].

It has been observed that constitutive NF-κB activation also occurs in long-term inflammation (i.e., in inflammatory bowel diseases (IBDs) patients), which induces the expression of cytokines, such as tumor necrosis factor (TNF)-α, interleukin (IL)-6, and other chemokines, supporting the inflammation-related tissue damage. Therefore, NF-κB may also contribute to the development of CRC by sustaining the ongoing inflammatory process in the intestinal tissue [69]. It has also been reported that both Akt and the Notch pathway may promote NF-κB effects by inhibiting the negative regulator IκB [70].

In 1971, Judah Folkman brought the concept that tumor growth beyond 2 mm is critically linked to angiogenesis, since it stimulates tumor development by providing nutrients and oxygen and also supplies growth factors that support tumor cell proliferation [71]. Moreover, cancer cell entrance into the systemic blood circulation is promoted by the immature neovasculature, and thus results in distant metastasis [72]. Vascular endothelial growth factor (VEGF) is the most broadly studied and best characterized angiogenic factor, released by almost all solid cancers, comprising CRC [72], even if, so far the serum VEGF relationship with the CRC is under debate [73,74,75].

Notch signaling plays a significant regulatory role in angiogenesis; unlike the VEGF antibodies, blocking the Notch–Dll4 axis increases the vascularization of the tumor [76]. However, the new vessels are abnormal with increased leakage, hyper-sprouting, loss of organization, and impaired tube formation, which leads to hypoxia and tumor shrinkage [77]. Recently, the Notch signaling inhibitor LY3039478 has been demonstrated to be comparatively safe and active as a single agent in preliminary studies in CRC patients [78]. Interestingly, a bispecific antibody targeting VEGF and Dll4 (ABL001/NOV1501/TR009), has been recently assessed in a phase 1 clinical trial of heavy chemotherapy or targeted therapy in pre-treated cancer patients, demonstrating higher anti-cancer effects in several human cancer xenografts than the VEGF-targeting antibody (bevacizumab—similar) and the Dll4-targeting monoclonal antibody alone [79].

Moreover, the activation of Notch signaling appears to be an early event in CRC development, since the process of ISC differentiation in the crypts is regulated at least in part by Notch receptors other than TGF-β signaling [80,81]. Notch expression in the primary stage of CRC is relatively higher than in the later stage [82]; indeed, its activation is induced by several factors, including Matrix metalloproteinases-9 (MMP9) under inflammatory conditions, which in turn promotes the EMT (in particular involving TGF-β), NF-κB and Wnt signaling pathways, thus affecting the stability of β-catenin, MAPK and the nutrient sensor kinase mTOR [83]. The pivotal role of Notch signaling in the onset of CRC suggests it as a promising candidate for diagnosis and may also contribute to the treatment resistance of CRC [80], but the unwanted side effects associated with Notch inhibitors usage are the major obstacle to entering clinics.

All these mentioned pathways represent fundamental paths that regulate development and may drive oncogenesis events. Several studies suggest that they are closely interconnected and understanding how they are interrelated is critical to the development of successful targeted therapies.

As already mentioned, sustained inflammation triggers the pathogenesis of CRC due to barrier disruption, and bacterial translocation resulting in inflammation and neoplastic transformation of colonic epithelial cells [84] predisposing to the cancer onset. For instance, Rokavec and colleagues reported a feedback loop among IL-6, Stat3, and miR34a, which can increase the invasiveness of CRC cells while the overexpression of IL-8 induces cancer growth and metastatization [85].

### 4.2. Involvement of Bile Acids in Colorectal Carcinogenesis

Studies back to the 1970s have highlighted that the risk for developing CRC was much higher among Japanese people who switched from a low-fiber to a high-fat ‘Western’ diet after immigration to the United States [86,87]. Since then, the prevalence of unhealthy dietary lifestyles has been linked to a dysregulation of gut microbiota [88], raising the incidence of CRC worldwide [89]. Specifically, both the dysbiosis of the resident gut microbiota (rather than simply certain pathogens), as well as the reduction in several helpful gut microbiota metabolites, such as short-chain fatty acids (SCFAs), have been shown to significantly alter the cancer risk or progression by causing immune response abnormalities [88].

Alexi N Archambault et. al. recently reported a risk prediction model for early onset CRC using 13 population-based studies (3486 cases and 3890 controls). This model incorporates a novel aggregate environmental risk score (ERS) and a recently expanded polygenic risk score (PRS) and allows for identifying individuals at differential relative and absolute risk for early onset CRC [90]. Indeed, CRC may be related to several factors: immunity, environment, dietary habits, and more general lifestyle, all involved in the microbiota composition [91]; recently it has been proposed that CRC is essentially a genetic disease, as well as a microbiological disease [88].

High-fat consumption stimulates the hepatic synthesis of BAs and their delivery to the colon, where they are metabolized by the microbiota into products with potential inflammatory and tumorigenic activity. BAs can also modify microbiota composition, leading to dysbiosis, thanks to their detergent properties [13], thus promoting a vicious circle with a tumor-promoting activity through the increased conversion to DCA and LCA [92]. As pointed out above, nutrients may play an important role in regulating BAs synthesis, which in turn regulates nutrient absorption and metabolic homeostasis. In fact, during the postprandial period, liver metabolism is highly active, and humans undergo fasting-to-refeeding cycles several times a day.

Some epidemiological studies have shown an association between high levels of fecal and serum BAs and CRC [93], above all the secondary BAs DCA and LCA, considered the “damaged bile acids” [94]. Detergency is clearly related to the BAs cytotoxicity, according to the BAs CMC (lower values correlate with higher detergency, as shown in Table 1: DCA > CDCA > CA > UDCA) (Figure 2). UDCA ranks as the less detergent and cytotoxic BA; LCA is the most hydrophobic BA but instead of a self-aggregation, it accumulates in a lipid domain, and this is consistent with the discoveries that UDCA, replacing endogenous BAs and LCA, on the contrary, often exerts a cytoprotective effect; LCA is mostly released into feces and often induces colonic carcinogenesis with DCA in CRC patients [13,95].

In 1940, DCA was firstly shown to be a carcinogen that induced CRC development in mice [96]; it is considered the most dangerous BA, since is reabsorbed in the colon thanks to its high hydrophobicity (while LCA is sulfonated and excreted in urines), and it is recycled with CA and CDCA to the liver. Indeed, the physiological recycled BAs pool (4 to 12 times a day) consists of ~3 g: ~40% CA, 40% CDCA, 20% DCA, and a trace amount of LCA.

Moreover, the hydrophobicity and detergency of DCA perturbate cell membranes by activating protein kinase C (PKC) and NADPH oxidase, which promote reactive oxygen species (ROS) and reactive nitrogen species (RNS) accumulation, causing oxidative stress that damages the DNA, disrupting the base excision repair pathway and able to induce NF-κB activation [40,97]. DCA can also activate EGFR to promote a hyperproliferative effect on colorectal mucosa, which is an indication of colorectal tumorigenesis in APC^min/+^ mice [98]. Moreover, Zeng et al. [99] discovered that DCA stimulated MAPK signaling in HCT116 cells through activation of SAPK/JNK1/2, p38 MAPK, and ERK1/2 pathways.

Despite the numerous studies that attributed the tumor-promoting function of BAs to DCA, studies performed in both human and animal models have also revealed that the dysregulation of CDCA with similar hydrophobicity and detergency, and its taurine-conjugated form (T-CDCA) synthesis, can lead to cancer-promoting effects in the liver and a consequent aberrant BAs metabolism [100]. Indeed, CDCA behaves as cytotoxic above its physiological concentration range. In a recent study, authors reported that CA also induces MMP-9 expression via ROS-dependent ERK-1/2, JNK AP-1, and p38-MAPK-activated NF-κB signaling pathways, which in turn stimulates cell invasion in human colon cancer cells [101].

These findings are not surprising, since the recent discovery that BAs are endogenous ligands of different receptors has given some mechanistic insight into the role of BAs in the regulation of gene transcription and signaling pathways [18], even if the underlying molecular mechanisms related to CRC did not remain fully elucidated. It is noteworthy that since BAs cannot induce tumor formation without a carcinogen/mutagen or a genetic mutation, they are proposed as tumor promoters but not as mutagenic agents themselves [97]. Consequently, BAs and especially secondary BAs have been proposed as tumor-promoting factors in CRC development [102].

### 4.3. Role of Bile Acid Receptors—Derived Signaling in Colorectal Cancer

As mentioned above, the digestive role of BAs in the intestine is to emulsify dietary lipids and cholesterol to facilitate their absorption. However, during the last two decades, extensive research has supported interesting evidence, showing that BAs not only are crucial in the digestive process but also play multiple important physiological functions besides their function as detergents.

The intestine is provided with complex bile acid-sensing processes that allow sophisticated coordination of different intestinal actions and regulate the crosstalk between the gut and other body organs. Seminal work by numerous investigators proposed the roles of BAs as signaling molecules, which can trigger cellular signaling pathways through the activation of specific receptors that modulate biological processes at low concentrations. BAs can activate both nuclear receptors and plasma membrane-associated receptors to elicit their physiological effects in the intestine [103].

Two major classes of intestinal receptors for BAs have been discovered in the gut: three nuclear receptors (NRs), farnesoid X receptor (FXR), pregnane X receptor (PXR)/steroid and xenobiotic receptor (SXR), vitamin D receptor (VDR), and the membrane Gαs protein-coupled receptor (GPCR) TGR5 [18,104,105]. Moreover, another bile acid-activated GPCR, called S1P2, was recently identified in hepatocytes and intestinal cells [106]. Table 2 reports the most relevant and updated studies describing new insights into bile acids-related signaling pathways in the onset of CRC.

#### 4.3.1. The Nuclear Receptors: FXR, VDR, and SXR/PXR

The nuclear BAs receptors play an important function in protecting against carcinogenic effects due to BAs themselves by activating transcriptional programs aimed at coordinating the maintenance of BAs uptake, detoxification, and basolateral secretion [103,107]. In 1995, the Evans lab described the isolation of an orphan receptor, which forms a heterodimer, with the retinoid X receptor (RXR), identifying farnesol and linked metabolites as strong complex activators [104]. In 1999, BAs were redefined as an unrecognized class of hormonal ligands because they were potent FXR ligands and farnesol, representing the chemical precursor to BAs [108]. Of multiple BAs-responsive NRs reported, the FXR is considered a master regulator in the gastrointestinal tract [104] It is noteworthy that two genes for FXR were discovered: FXRα and FXRβ. The role of FXRβ is not well understood in mice, and it is proposed to be a pseudogene in humans so in this review we refer just to FXRα, simply named FXR [103].

**Figure 5:**
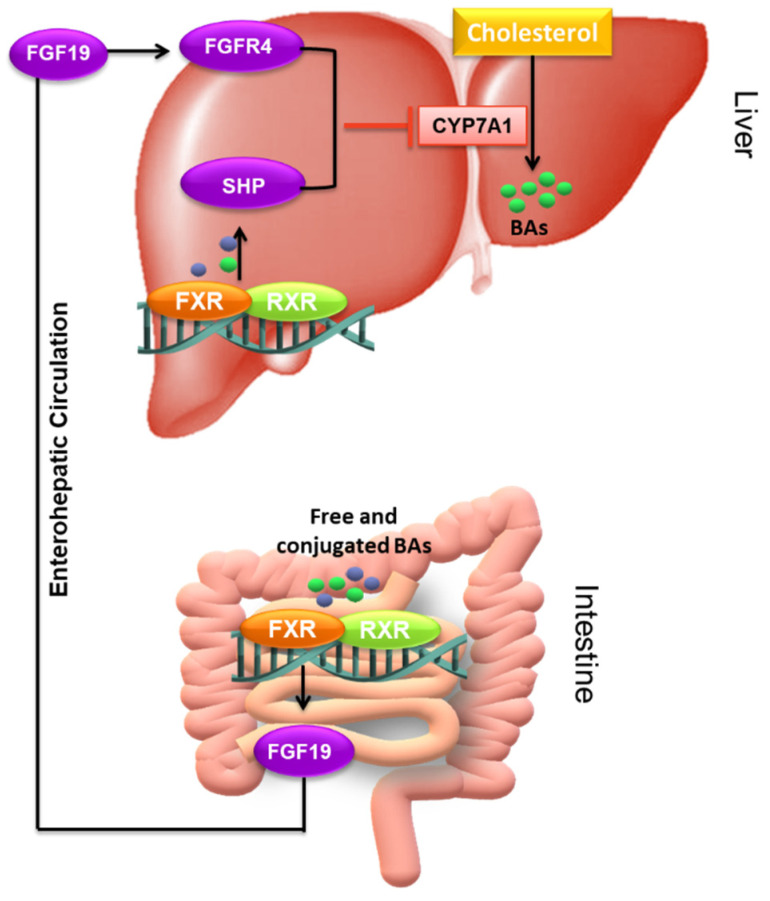
Schematic representation of the role of FXR in the regulation of BAs synthesis. BAs that travel back in the enterohepatic circulation (EHC) activate hepatic and intestinal FXR to regulate genes important for BAs synthesis. The activation of FXR in the gut induces human fibroblast growth factor (FGF)-15/19 (15 in mice and 19 in humans, respectively) expression, which is secreted through the circulation to the liver. Here, it binds and activates the FGFR-4, which in turn inhibits CYP7A1 expression. Moreover, the activation of hepatic FXR represses BAs synthesis by reducing CYP7A1 expression via SHP induction. The figure is original and created with BioRender.com.

FXR is highly expressed in the liver and intestine (mainly in the ileum) and its activation occurs in the presence of free and conjugated BAs. In vitro studies have consistently shown a rank order for the affinity of BAs to bind FXR: CDCA > DCA > LCA > CA (CDCA: EC50 =~10 μM) [109]. Briefly, cellular uptake of CDCA through ASBT promotes FXR in ileal epithelial cells, leading to its heterodimerization with RXR, translocation to the nucleus, and binding to FXR responsive element (FXRE). The activation of FXR induces human fibroblast growth factor (FGF)-15/19 (15 in mice and 19 in humans, respectively) expression, which is released through the circulation to the liver. Here, it binds and activates the tyrosine kinase receptor fibroblast growth factor (FGFR)-4, which in turn induces the phosphorylation cascades of both ERK and JNK pathways, ultimately inhibiting CYP7A1 expression hence BAs synthesis (Figure 5) [110,111]. In the liver, FXR inhibits BAs synthesis also through a feedback mechanism that involves the small heterodimer partner (SHP). Upon activation by its ligand, FXR induces the transcription of SHP, which in turn directly interacts with the liver receptor homologous-1 (LRH-1), a competent transcription factor for CYP7A1, inhibiting its transcriptional activity, and thus repressing CYP7A1, the rate-limiting enzyme in de novo bile acid synthesis (Figure 5) [112]. This mechanism works synergistically with the intestinal FXR-FGF15/19-dependent one, conferring transcriptional repression of CYP7A1 and, as a result, mediating the suppression of hepatic BAs synthesis (Figure 5).

FXR regulates not only the BAs pool but also BAs uptake and export systems by inducing some binding proteins and transporters, i.e., IBABP and OSTα/OSTβ or by inhibiting other ones, such as NTCP and ASBT [95]; so, any alteration of normal intestinal FXR expression and activity associated with compromised BAs homeostasis leads to the augmented production of cytotoxic, proinflammatory, and pro-tumoral secondary BAs [34]. Thus, BAs synthesis is under negative-feedback control through the different enterohepatic FXR-dependent signaling axis. During cholestasis, when high levels of BAs are present in the liver, FXR also induces OSTα/β to allow BAs to spill over from the liver in the systemic circulation for their final urine excretion. Under these pathological conditions, FXR also induces the expression of phase I (CYP3A4/CYP3A11) and phase II (SULT2A1 and UGTB4) enzymes to turn BAs into more hydrophilic and less toxic molecules that are efficiently eliminated [95].

Given the central role of FXR in the preservation of BAs concentrations within a physiological range, which prevents BA-induced cytotoxicity,, the loss of FXR would be expected to be related to a pro-tumorigenic phenotype [113]. De Gottardi et al. gave the first evidence of the variation of FXR expression in CRC, observing that in more than 60 patients FXR mRNA was downregulated in colorectal adenoma and carcinoma, whereas IBABP significantly increased. Simultaneously, they discovered that undifferentiated colon carcinoma cell line SW480 cells lacking FXR display aggressive growth potential, while significant FXR mRNA amounts are detected in two less aggressive cell lines, Caco-2 and HT-29 cells [114]. Moreover, immunohistochemical analysis of 159 stage I-IV CRC samples, 32 polyps, and 238 normal samples showed a loss of FXR expression in the majority (94%) of CRC samples, and a marked reduction in FXR expression in precancerous polyps, compared with normal colon tissues. Moreover, FXR negatively correlated with K-Ras signaling activation [115]. As previously reported, hyperactivation of the Wnt/β-catenin pathway is believed to be the initiating and driving event related to CRC and mechanistic findings highlighted that FXR exerted its tumor suppressor functions by antagonizing Wnt/β-catenin signaling. Indeed, there is a reciprocal relationship between FXR and β-catenin, since the loss of β-catenin increases the transcriptional activation of SHP by FXR [116]. Li et al. found that FXR agonist obeticholic acid (OCA) inhibits the development and invasion of CRC by attenuating the EMT induced by Wnt/β-catenin and suppressing the activity of the Jak2/Stat3 pathway in human colon cancer cells [117]. The existing link between FXR deficiency, Wnt/β catenin activation, and intestinal carcinogenesis was also confirmed in animal models. In mice with APC gene silencing mutation (Apc^min/+^) or those treated with the colon chemical carcinogen azoxymethane (AOM)/dextran sulfate sodium (DSS), FXR loss promotes intestinal tumor progression and an increased adenoma size, which leads to early mortality, while the transgenic overexpression of FXR in gut cells reduces tumor growth and development [113,118]. It was also demonstrated that treatment with an FXR agonist inhibits the aberrant Lgr^5+^ ISCs proliferation and curtails CRC progression in an APC^min/+^ mouse model. In line with these observations, Farhana et al. revealed that DCA is able to induce ISCs proliferation by modulating muscarinic 3 receptors (M3R) and Wnt/β-catenin signaling, presumably acting through FXR [119].

Taken together, these data suggest that the loss of FXR expression promotes the transcriptional activity of β-catenin, whereas FXR activation results in the opposite effect in both human colon cancer mouse models and cell lines. Nevertheless, FXR functions as a tumor suppressor in CRC, at least in part by antagonizing Wnt/β-catenin signaling [116,120].

Notably, in colon cancer cells, the constitutive activation of FXR can suppress colonic epithelium proliferation and induce the expression of a pro-apoptotic network of genes (p21, TNFα and the FAS receptor) while repressing anti-apoptotic genes, such as Bcl-2 [121], showing the high predisposition to intestinal adenocarcinoma due to decreased negative regulation of the FXR–FGF19 axis. So, there is a direct association between intestinal cancer incidence and FXR expression; indeed, the ileum has the greatest level of FXR expression [121], and it gradually decreases from the terminal ileum to the sigmoid colon, thus explaining the rare occurrence of carcinoma in the ileum and the highest incidence of carcinoma in the distal portions of the colon, which have low FXR expression levels [122].

The process behind the silencing of FXR in CRC may lie in its DNA methylation caused by mutated APC that, in turn, leads to reduced expression of SHP and IBABP and induced cyclooxygenase-2 (COX-2) and c-Myc [123] and also by K-Ras signaling activation [115,124]. Moreover, in inflamed mouse colon tissue, the activity of FXR can be inhibited by the activation of NF-κB or peroxisome proliferator-activated receptor α (PPARα)–glucuronosyltransferases (UGT) axis activation, which was positively associated with the development of CRC [95] and negatively associated with the TGF-β-dependent pathway activation [125].

K-Ras is a frequently mutated oncogene in CRC; its diverse mutations promote the activation of the MAPK pathway and cause spontaneous tumor development [126], and the aberrant activation of the PI3K/Akt/mTOR pathway strongly attenuates the efficacy of MAPK suppression [127]. It is reported that inhibition of K-Ras signaling in human colon cancer cells resulted in a strong increase in FXR levels [115] while FXR downregulation has been negatively related with PI3K signaling in human colon cancer [128].

There is a mutual antagonism between FXR and NF-κβ too because not only does FXR promote inflammation inhibition but also, in different model systems, its activation is inhibited by pro-inflammatory stimuli (TNF-α and IL-1β) [124]. Firstly, the pro-inflammatory cytokine TNFα decreased FXR expression in enterocyte-like differentiated HT29 cells. This finding was confirmed in ex vivo mice ileal specimens of WT and FXR-ko. Furthermore, in mice with severe intestinal inflammation induced by DSS, the expression of FXR target genes IBABP and FGF15 was similarly reduced in the ileum as well as in the colon [129].

FXR is also involved in modulating other CRC-related pathways; for instance, the knockdown of FXR induces the expression of several EMT markers, such as vimentin, snail, slug, fibronectin, and MMP-9, while suppressing E-cadherin and zonula occludens-1 (ZO-1) [116]. On the other hand, FXR activation inhibits the expression of MMP-7, an important contributor to colon cancer metastasis [130].

FXR also takes part in the regulation of antibacterial defense by controlling the expression of inducible nitric oxide synthase (iNOS), IL-18, angiogenin1 (ANG1), and carbonic anhydrase 12 (CAR12) [131], thus indirectly modulating the gut microbiota composition.

All these data suggest there is an inverse correlation between the FXR expression levels and CRC progression and malignancy. So, the FXR gene is supposed to have some characteristics of a tumor suppressor in colon cancer; in this context, the restoration of basal FXR expression might slow or prevent the progression of colon cancer allowing it to be a potential prognostic and target for the treatment of CRC.

Recently, it has been discovered that not just FXR, but also the nuclear BAs receptor vitamin D receptor (VDR), the rodent pregnane X receptor (PXR), and its human homolog steroid and xenobiotic receptor (SXR) play an important role in protecting against carcinogenic effects of BAs by activating transcriptional programs aimed at coordinating the control of BAs uptake, detoxification, and basolateral secretion [132]. VDR and PXR/SXR are receptors for LCA and its metabolites (3-oxoLCA and iso-allowed), reputed as one of the few toxic endobiotic [133]. The NRs activation induces the production of CYP3A in the liver and intestine to catabolize LCA excess, thereby giving protection against its toxicity. VDR and PXR/SXR also contribute to the enterohepatic BA–FGF15/19–CYP7A1 negative feedback loop to control hepatic BAs synthesis [104]. These findings suggest a model explain how the enteric system could protect itself from the potentially harmful effects of LCA.

Vitamin D deficiency is recognized as a general risk factor for CRC; when vitamin D binds to VDR it behaves as a transcription factor for many genes, exerting profound antimitogenic and differentiating effects on many normal and malignant cells, including colon cancer cells [134,135]. Concordantly, VDR expression is enhanced during the early stages of colon cancer but, during late colon cancer progression, it is downregulated, probably due to the upregulation of the transcription factors SNAI (snail family zinc finger) 1 and 2 [136,137]. So, a high level of VDR expression is recognized as an early favorable prognosis for CRC [138].

The protection given by VDR activation may become compromised when the detoxification pathway is overwhelmed (e.g., by increased levels of LCA due to sustained high-fat diets) or under pathologies of vitamin D deficiency (e.g., rickets/osteomalacia) [139]. Indeed, after binding to VDR, both LCA and vitamin D may stimulate a feed-forward catabolic pathway with a consequent increasing of CYP3A expression and LCA detoxification [140]. CYP3A in the liver metabolizes more than 50% of all administered drugs [141], thereby also reducing the efficacy of chemotherapeutic agents [142], and thus its modulation needs careful evaluation. Fecal microbiota analysis showed that lacking VDR is linked with dysbiosis and susceptibility to CRC via reducing JAK/STAT signaling and dampening inflammatory reactions [143].

Recently, the strength of the causal link between vitamin D and colon cancer has been called into question; He et al. recently reported that in over 10,000 colorectal cancer cases and 30,000 controls there was no evidence for a relationship between 25(OH)D3 levels and colorectal cancer risk [144]. Irving et al. demonstrated in both rat and mouse models of colon cancer that vitamin D supplementation does not protect against intestinal tumor development; it instead enhances tumorigenesis in the colon of the rat [145,146]. Moreover, the same group recently reported that loss of VDR expression alone did not induce tumorigenesis, even in animals exposed to the carcinogenic molecule sodium dextran sulfate [147]. Moreover, in Apc^Pirc/+^ rats, VDR loss did not enhance tumor multiplicity, growth, or progression in the colon, thus supporting previous findings that vitamin D itself does not play a role in colon cancer development or progression. To sum up, there is not a clear association between vitamin D and colon cancer risk; human studies are needed to better define the link between Vitamin D, VDR, and colon cancer.

Likewise, the human xenobiotic receptor SXR and its rodent homolog PXR are nuclear BAs receptors expressed in the intestine and liver that when activated induce a response that detoxifies BAs [148]. SXR promotes BAs detoxification through the activation of BAs metabolizing enzymes and transporters; indeed, it binds the promoter and upregulates the expression of several CYPs (CYP3A, CYP2B, and CYP2C), the UDP-glucuronosyltransferase 1A1 (UGT1A1), as well as the xenobiotic transporters multidrug resistance 1 (MDR1) and organic anion transporter 2 [149]. Upon the chemotherapeutic irinotecan treatment, PXR/SXR is translocated into the nucleus, binds to RXR, and the heterodimer interacts with the promoter of the CYP3A4 gene to induce its expression in colon cancer cell lines. Therefore, cells overexpressing the PXR/SXR appear to be significantly less sensitive to irinotecan, possibly due to an enhanced expression of CYP3A4 [150]. As a result, PXR/SXR may play a central role in the induction of drug resistance [151]. Moreover, a high PXR level is associated with poor prognosis in stage II/III colon cancer patients treated with chemotherapy. Indeed, PXR is specifically present in CSCs, where it leads the expression of genes involved in self-renewal and chemoresistance. PXR silencing increased the chemosensitivity of human colon CSCs, reduced their self-renewal and tumor-initiating potential, and significantly delayed tumor recurrence in mice following chemotherapy [152]. In this context, PXR may be considered a clinically druggable Achilles’ heel for CSCs in CRC. On the other hand, in both human colon cancer cells and normal mouse colon epithelium, PXR/SXR protects against oxidant-induced apoptosis [153] and inhibits the proliferation and tumorigenicity of HT29 colon cancer cells by controlling the cell cycle at the G0/G1 cell phase by regulating p21WAF1/CIP1 and E2F/Rb pathways [154].

**Table 2:** Summaries of the main contributions of previous studies describing new insights into BAs-related signaling pathways in the onset of CRC.

Pathway	Bile Acid	BAs Receptor	References
**NF-κB signaling**	DCA	*Not specified*	[92]
	CA	*Not specified*	[96]
	CDCA	TGR5	[153]
	*Not specified*	FXR	[90,119,124]
	*Not specified*	TGR5	[149,154]
**EGFR axis**	DCA	*Not specified*	[93]
**MAPK signaling**	DCA	*Not specified*	[94]
	CA	*Not specified*	[96]
	*Not specified*	FXR	[105,106]
	*Not specified*	*Not specified*	[92]
**K-Ras signaling**	*Not specified*	FXR	[110]
**Wnt/β-catenin signaling**	OCA	FXR	[112]
	DCA	FXR	[114]
	*Not specified*	FXR	[108,111,113,115]
**JAK2/STAT3 signaling**	OCA	FXR	[112]
	*Not specified*	*Not specified*	[138]
	CDCA	TGR5	[153]
	*Not specified*	TGR5	[154]
**PI3K signaling**	*Not specified*	FXR	[123]

Altogether, these results reveal a complex network of interactions, suggesting that the PXR/SXR pathway promotes the expression of detoxification genes, thereby leading to enhanced drug resistance, but, on the other hand, their induction gives an anti-proliferative effect in colon cancer cells.

In summary, FXR is downregulated in both mouse and human models of CRC when the tissues are progressing from normal intestinal epithelia to dysplastic lesions, indicating a therapeutic and/or diagnostic potential for these transcription factors in CRC. However, the role of VDR and PXR/SXR in CRC is still controversial, and more studies are necessary to clarify their activities in colon cancer cells.

#### 4.3.2. The Membrane Receptors: TGR5 and S1PR2

The BA receptor TGR5, discovered later than FXR, was first recognized as a regulator of energy and bile acids homeostasis, as well as glucose metabolism [155]. It is a membrane G-protein coupled receptor (GPCR) composed of seven transmembrane domains; upon binding of its ligand in the extracellular space, it transduces the extracellular signal to intracellular downstream cascades by activating the adenylyl cyclase cyclic adenosine monophosphate (cAMP) signaling pathways [156,157].

Different conjugated and unconjugated BAs are ligands for TGR5. In vitro studies have highlighted that the unconjugated bile acid rank order for TGR5 activation is: LCA > DCA > CDCA > CA > UDCA (EC50 of LCA: 35 ± 5 nM) [158]. TGR5 mRNA was identified in several tissues, such as the small and large intestine, stomach and liver; the receptor expression is reported in various cell types, including gallbladder epithelial cells (cholangiocytes), gallbladder smooth muscle cells, Kupffer cells, intestinal L cells, pancreatic β cells, skeletal muscle cells, nerve cells, and brown adipocytes [159].

Despite its physiological role in integrating intestinal homeostasis, intestinal barrier integrity and glucose metabolism are well defined; TGR5 also seems to be involved in other cell signaling pathways, such as NF-κB, the central transcriptional regulator of inflammatory and immune responses, Akt, and ERK. Indeed, mice lacking TGR5 develop altered colonic histopathology, which results in severe alteration in the distribution and maturation of mucous cells and the disruption of the molecular architecture of colonic tight junctions [160]. Since tight junction alterations are a well-recognized hallmark of intestinal inflammation, these data support the view that TGR5 provides regulatory signals to intestinal epithelial cells. Furthermore, TGR5 exerts anti-inflammatory functions by antagonizing TNFα and NF-κB -dependent induction of pro-inflammatory cytokines also in macrophages; indeed, in TGR5 knockout mice macrophages, the mRNA levels of various pro-inflammatory genes targeted by NF-κB (iNOS, interferon-inducible protein, and IL-1α) are greater than to those in macrophages from WT mice [161]. In line with these data, in the macrophage RAW264.7 cell line, TGR5 activation inhibited NF-κB activation via the cAMP signaling pathway [162].

Continuous inflammation was involved in the CRC pathogenesis due to barrier disruption, and bacterial translocation resulted in the inflammation and neoplastic transformation of colonic epithelial cells [163]. TGR5 activation by UDCA and LCA may also exert anti-inflammatory reactions through TLR4 activation or by reducing pro-inflammatory cytokines in the colon, which can decrease the frequency of developing CRC [164]. Moreover, the release of endogenous BAs in the intestinal lumen triggers ISCs renewal and proliferation through TGR5–SRC–YAP axis activation and drives regeneration in response to injury [47].

The ability of TGR5 to lower the levels of pro-inflammatory cytokines in intestinal cells and macrophages has opened new insights into the modulatory role of BAs in diseases where inflammatory processes play a central role, such as CRC; however, its involvement in tumors has to be clarified since TGR5 seems to have different actions in different cell and tissue background.

For instance, Guo et al. (2015) and Chen et al. (2012) observed that TGR5 acts as a suppressor of cancer cells migration and proliferation in gastric [165] and liver [166] cancers by antagonizing Stat3 signaling, which is an important transcriptional factor that regulates several pathways involved in tumorigenesis. Importantly, in the mouse model of liver carcinogenesis, TGR5 knockout mice had a higher incidence of hepatocellular carcinoma than WT mice, and Stat3 phosphorylation, which is substantially associated with an increased tumor progression, was 2-fold higher than in WT mice liver [166].

Many reports have shown the Stat3 activation in various tumor cell lines, including colon cancer cells [167]; however, the direct implication of TGR5 in colonic carcinogenesis is still under debate [156]. A recent study performed by Zhang et al. has demonstrated that CRC tissues have a lower TGR5 expression when compared with normal colon and rectum tissue, and this decrease is linked to a poor prognosis after considering the overall survival status [168]. Moreover, they showed that UDCA treatment, which is already known for its anti-cancer effects, raised TGR5 expression at both mRNA and protein levels in HCT116 and SW480 cells. In AOM/DSS-induced primary CRC mice models, UDCA, acting as a TGR5 agonist, downregulated the YAP pathway, which is linked to tumor growth, reducing the CRC progression, and thus demonstrating a protective effect. These in vivo results were in line with in vitro results and indicated that UDCA repressed the CRC cell proliferation by inhibiting YAP and activating TGR5. Indeed, endogenous BAs in the intestinal lumen trigger ISCs renewal and proliferation through TGR5–SRC–YAP axis activation and drives regeneration in response to injury [47].

So, TGR5 activation likely promotes intestinal healing via a dual mechanism encompassing an anti-inflammatory activity and a pro-regenerative program in epithelial cells and could be further explored in CRC clinical settings.

Sphingosine-1-phosphate receptor 2 (S1PR2 or S1P2) is a G protein-coupled receptor for sphingosine-1-phosphate (S1P) expressed in the ileum and colon, activated also by glycine and taurine conjugated primary bile acids [169]. In normal colonic crypts, S1PR2 increases expression along with intestinal epithelial cells differentiation, but not in intestinal stem cells, and reduces intestinal tumorigenesis by promoting epithelial differentiation, preventing the expansion of stem cells and breaking their malignant transformation [170]. Indeed, the loss of S1PR2 induces the expansion of intestinal stem cells in colon cancer cells and, clinically, S1PR2 expression was lost in 33% of adenocarcinomas with stage II/III pT3-T4 and significantly decreased in 55% [159]. Moreover, preliminary data suggest that S1PR2 deletion exacerbates intestinal inflammation caused by DSS in mice [171]. These data are still under debate since S1PR2 expression in CRC patients has scarcely been investigated. Indeed, Shida et al. et al. showed a varied expression profile of this receptor without any trend along the CRC tissues analyzed [172], while Uranbileg et al. showed an increased expression of S1PR2 in tumor samples compared to normal tissue samples [173].

S1PR2 activated by TCA stimulates the sphingosine kinase (SphK2) expression and activity by increasing the conversion of sphingosine into S1P, leading to increased lipid and sterol metabolism in the liver [174]. Thus, conjugated BAs are also key players in S1P signaling via SphK2 induction, as well as S1PR2. Consistent with these findings, both SphK2 and S1PR2 knockout mice are susceptible to the diet-induced fatty liver [175]. Interestingly, nuclear S1P produced by either induction of SphK2 or inhibition of S1Plyase, binds to histone deacetylases (HDAC) 1 and 2, thereby increasing histone acetylation and up-regulating the expression of metabolic genes. Through such HDAC inhibitory process, sphingosine regulates apoptosis and metabolism [176].

To date, however, we have only a few studies investigating the potential therapeutic effects of S1PR2 in CRC, even if preliminary data identify it as a promising candidate tumor suppressor gene in CRC.

## 5. Conclusions

Since the CRC incidence and mortality rates are expected to grow in the near future, understanding the molecular and cellular mechanisms underlying CRC becomes pivotal. The evidence reviewed here indicates that perturbations of BAs synthesis and composition are related to colorectal oncogenic signals, even if a clear link between BAs receptors, located in the gut, and CRC has not yet been fully elucidated. Diet and the consequent gut microbiota profile are most likely the key environmental drivers that mediate and confer BAs-associated tumorigenic activity. Indeed, a high-fat diet-associated BAs level impact gut microbiota composition favoring increased levels of 7α-dehydroxylation bacteria, which stimulate secondary BA formation with tumor-promoting activity.

Further studies are required to demonstrate or exclude a causal correlation between BAs receptor levels in the gut and CRC onset. This would allow researchers to better understand which patients would obtain not just the greatest preventative benefit from reducing their secondary BAs levels, with diet and/or with specific drugs, but also to define subjects with high-CRC risk for whom finding biomarkers, such as BAs receptors profile in the gut, is an urgent clinical need, especially for recurrences. Unfortunately, the overall 5-year survival rate of CRC is approximately 60% and after curative treatment, 30% to 40% of the patients develop recurrent disease [177].

Right now, BAs profile analysis and the characterization of microbe–host interactions implicated in bile acid metabolism can be considered diagnostic biomarkers for CRC prevention. However, it is not yet possible to conclude whether a dysregulated intestinal BA receptors profile or their derived signaling pathways detrimentally affect the risk of developing CRC.

The application of specific drugs, probiotics, and their products, as well as lifestyle changes may become effective interventions. We look forward to furthering research and exploration to provide further mechanisms for the occurrence and development of CRC and new diagnostic directions in the near future.

## Figures and Tables

**Table 1:** BAs physicochemical properties. BAs hydrophobic scale: CA < UDCA = CDCA = DCA < LCA. CA is the most hydrophilic and LCA as the most hydrophobic BA. BAs detergency rate: DCA > CDCA > CA > UDCA. So, CA, is even less hydrophobic than UDCA, which is more detergent. The water solubility of LCA is less than 0.1 µM and does not reach a concentration to form micelles even when ionized [22,23,24,25].

	R1	R2	R3	R4	LogP_A^-^_	CMC (mM in 0.15 M Na^+^)
**CA**	OH	OH (α)	OH	OH	1.1	9
**CDCA**	OH	OH (α)	H	OH	2.3	3.2
**UDCA**	OH	OH (β)	H	OH	2.2	10
**DCA**	OH	H (α)	OH	OH	2.7	3
**LCA**	OH	H (α)	H	OH	5.5 (est)	-*
**G-CA**	OH	OH (α)	OH	Glycine	−0.4	8
**T-CA**	OH	OH (α)	OH	Taurine	−0.5	4
**G-CDCA**	OH	OH (α)	H	Glycine	0.5	2
**T-CDCA**	OH	OH (α)	H	Taurine	0.9	3
**G-UDCA**	OH	OH (β)	H	Glycine	0.2	4
**T-UDCA**	OH	OH (β)	H	Taurine	1.1	6
**G-DCA**	OH	H (α)	OH	Glycine	0.8	2
**T-DCA**	OH	H (α)	OH	Taurine	0.7	2.5
**G-LCA**	OH	H (α)	H	Glycine	3.5 est)	-*
**T-LCA**	OH	H (α)	H	Taurine	3 (est)	-*

* do not self-aggregate due to their low water solubility; est: estimated; LogP_A^-^_: octanol/water partition coefficient of the ionized form; CMC: critical micellar concentration of the bile salt in Na^+^ 0.15 M. T: taurine. G: glycine.
